# Computed tomography assessment of PEEP-induced alveolar recruitment in patients with severe COVID-19 pneumonia

**DOI:** 10.1186/s13054-021-03477-w

**Published:** 2021-02-24

**Authors:** Lorenzo Ball, Chiara Robba, Lorenzo Maiello, Jacob Herrmann, Sarah E. Gerard, Yi Xin, Denise Battaglini, Iole Brunetti, Giuseppe Minetti, Sara Seitun, Antonio Vena, Daniele Roberto Giacobbe, Matteo Bassetti, Patricia R. M. Rocco, Maurizio Cereda, Lucio Castellan, Nicolò Patroniti, Paolo Pelosi, GECOVID (GEnoa COVID-19) group, Lorenzo Ball, Chiara Robba, Lorenzo Maiello, Denise Battaglini, Iole Brunetti, Giuseppe Minetti, Sara Seitun, Antonio Vena, Daniele Roberto Giacobbe, Matteo Bassetti, Lucio Castellan, Nicolò Patroniti, Paolo Pelosi, Angelo Gratarola, Maurizio Loconte, Alexandre Molin, Giulia Orefice, Francesca Iannuzzi, Federico Costantino, Dario Battioni, Giulio Bovio, Gerolama Buconte, Alessandro Casaleggio, Giuseppe Cittadini, Luca Dogliotti, Veronica Giasotto, Maria Pigati, Elena Santacroce, Federico Zaottini, Chiara Dentone, Lucia Taramasso, Laura Magnasco, Alberto Valbusa, Matilde Bastianello

**Affiliations:** 1 Department of Surgical Sciences and Integrated Diagnostics (DISC), University of Genoa, Viale Benedetto XV 16, Genoa, Italy; 2 Anesthesia and Intensive Care, Ospedale Policlinico San Martino, IRCCS Per L’Oncologia e le Neuroscienze, Genoa, Italy; 3 Department of Biomedical Engineering, Harvard Medical School, Boston, MA USA; 4 Department of Radiology, Harvard Medical School, Boston, MA USA; 5 Department of Radiology, Perelman School of Medicine, University of Pennsylvania, Philadelphia, PA USA; 6 Radiology Department, Ospedale Policlinico San Martino, IRCCS Per L’Oncologia e le Neuroscienze, Genoa, Italy; 7 Infectious Diseases Unit, Ospedale Policlinico San Martino, IRCCS Per L’Oncologia e le Neuroscienze, Genoa, Italy; 8 Department of Health Sciences (DISSAL), University of Genoa, Genoa, Italy; 9 Laboratory of Pulmonary Investigation, Carlos Chagas Filho Institute of Biophysics, Federal University of Rio de Janeiro, Rio de Janeiro, Brazil; 10 Department of Anesthesiology and Critical Care, Perelman School of Medicine, University of Pennsylvania, Philadelphia, PA USA

**Keywords:** COVID-19, ARDS, Respiratory system mechanics, Mechanical ventilation, CT scan

## Abstract

**Background:**

There is a paucity of data concerning the optimal ventilator management in patients with COVID-19 pneumonia; particularly, the optimal levels of positive-end expiratory pressure (PEEP) are unknown. We aimed to investigate the effects of two levels of PEEP on alveolar recruitment in critically ill patients with severe COVID-19 pneumonia.

**Methods:**

A single-center cohort study was conducted in a 39-bed intensive care unit at a university-affiliated hospital in Genoa, Italy. Chest computed tomography (CT) was performed to quantify aeration at 8 and 16 cmH_2_O PEEP. The primary endpoint was the amount of alveolar recruitment, defined as the change in the non-aerated compartment at the two PEEP levels on CT scan.

**Results:**

Forty-two patients were included in this analysis. Alveolar recruitment was median [interquartile range] 2.7 [0.7–4.5] % of lung weight and was not associated with excess lung weight, PaO_2_/FiO_2_ ratio, respiratory system compliance, inflammatory and thrombophilia markers. Patients in the upper quartile of recruitment (recruiters), compared to non-recruiters, had comparable clinical characteristics, lung weight and gas volume. Alveolar recruitment was not different in patients with lower versus higher respiratory system compliance. In a subgroup of 20 patients with available gas exchange data, increasing PEEP decreased respiratory system compliance (median difference, MD − 9 ml/cmH_2_O, 95% CI from − 12 to − 6 ml/cmH_2_O, *p* < 0.001) and the ventilatory ratio (MD − 0.1, 95% CI from − 0.3 to − 0.1, *p* = 0.003), increased PaO_2_ with FiO_2_ = 0.5 (MD 24 mmHg, 95% CI from 12 to 51 mmHg, *p* < 0.001), but did not change PaO_2_ with FiO_2_ = 1.0 (MD 7 mmHg, 95% CI from − 12 to 49 mmHg, *p* = 0.313). Moreover, alveolar recruitment was not correlated with improvement of oxygenation or venous admixture.

**Conclusions:**

In patients with severe COVID-19 pneumonia, higher PEEP resulted in limited alveolar recruitment. These findings suggest limiting PEEP strictly to the values necessary to maintain oxygenation, thus avoiding the use of higher PEEP levels.

## Introduction

Over the last months, the global pandemic from coronavirus disease 2019 (COVID-19) has posed important challenges to intensive care unit (ICU) physicians [1, 2]. A significant proportion of COVID-19 patients develop severe hypoxemic respiratory failure requiring invasive mechanical ventilation [2, 3]. Although COVID-19 meets the clinical criteria for acute respiratory distress syndrome (ARDS) [4], peculiar pathophysiological features [5] and phenotypes have been identified in this disease [6]. In COVID-19 patients, chest computed tomography (CT) findings typically include ground glass opacities overlapping with areas of lung consolidation, not always reflecting the severity of gas-exchange impairment [7]. In this context, severe hypoxemia might be related not only to loss of aeration, but also to highly perfused ground-glass areas [8, 9]. In COVID-19 patients with high respiratory system compliance and low ventilation-perfusion ratio (${\dot{V}}_{A}/\dot{Q}$), hypoxemia is primarily due to the ${\dot{V}}_{A}/\dot{Q}$ mismatch, which is more related to lung perfusion regulation impairment than to an increase in non-aerated tissue; therefore, lung recruitability is probably low [8, 9].

To date, no specific recommendations are available concerning the optimal PEEP levels in invasively ventilated COVID-19 patients [10]. It has been suggested that COVID-19-associated ARDS might share common features with ordinary ARDS [11], in which the use of higher PEEP levels is frequently advocated [12], even if this strategy is not supported by the findings of recent trials [13]. Nevertheless, the pathophysiology of COVID-19 seems to differ from that of ARDS [9] and limited physiological data is available on PEEP response in severe COVID-19 patients. We therefore conducted an observational study with the aim to investigate the effect of two levels of PEEP (8 and 16 cmH_2_O) on alveolar recruitment in severe COVID-19 patients. We hypothesized that the PEEP increase resulted in limited alveolar recruitment in COVID-19 patients.

## Methods

This cohort study was carried out in a university-affiliated hospital in Genoa, Italy. The ethics review board approved the protocol of the study (Comitato Etico Regione Liguria, protocol n. 163/2020) and the need for written informed consent was waived for retrospectively collected data. According to local regulations, consent was delayed after discharge for prospectively collected data in unconscious patients.

### Patient inclusion, data collection and clinical management

This study included all critically ill, invasively ventilated COVID-19 patients admitted from February 29th to May 15th, 2020 that underwent a two-PEEP CT scan on clinical indication. All patient had a positive polymerase chain reaction on nasopharyngeal swab specimens and fulfilled clinical criteria for severe COVID-19 pneumonia [14, 15]. Clinical data were collected retrospectively from the electronic medical records. The Additional file 1 reports details on the two-PEEP CT clinical indications, image acquisition technique and analysis protocol. Patients were ventilated targeting tidal volumes of 6 mL per kg of predicted body weight, but increases were tolerated based on the driving pressure. The respiratory rate was titrated to maintain pH above 7.25. The clinical PEEP level was decided by the treating physician, aimed at maintaining PaO_2_ > 60 mmHg with the lowest possible plateau pressure.

### Gas exchange and respiratory mechanics assessment

Blood gas analyses and ventilation parameters were collected in all patients on the day of the CT scan. The ventilatory ratio [16] was computed as:

$${\text{Ventilatory}}\;{\text{Ratio}} = \frac{{{\text{minute}}\;{\text{ventilation}} \left( {\text{ml/min}} \right) \times {\text{Pa}}_{{{\text{CO}}_{2} }} \left( {{\text{mmHg}}} \right)}}{{{\text{predicted}}\;{\text{body}}\;{\text{weight}} \left( {{\text{kg}}} \right) \times 100 \times 37.5\;{\text{ mmHg}}}}$$

The ventilatory ratio is an estimate of ventilation impairment and is known to correlate with physiologic dead-space fraction in COVID-19 patients [17]. A sub-group of patients underwent a PEEP test at 8 and 16 cmH_2_O at a FiO_2_ of 1.0 to estimate venous admixture and at a FiO_2_ of 0.5, the latter value being arbitrarily chosen to explore the effects of FiO_2_ changes on oxygenation. All four possible PEEP/FiO_2_ combinations were tested. Blood gas analyses and respiratory mechanics were assessed within 2 h from the CT scan and included estimation of venous admixture based on arterial and central venous blood gas samples (details in Additional file 1).

### Protocol for two-PEEP CT acquisition and analysis

Patients received non-contrast chest CT scan at PEEP 8 cmH_2_O during expiratory breath-hold, then PEEP was increased to 16 cmH_2_O and the CT scan repeated after around 1 min of ventilation with PEEP 16 cmH_2_O and unchanged tidal volume, resulting in plateau pressures ranging from 25 to 35 cmH_2_O. No recruitment maneuver was performed. Lung parenchyma and vessel segmentations were obtained using multi-resolution convolutional neural networks [18], followed by manual refinement if necessary. Also, three regions of interests (ROIs) of equal lung tissue weight [19, 20] were obtained along the ventral-dorsal and craniocaudal axes. Lung was divided into hyper-, normally, poorly, and non-aerated compartments, according to conventional thresholds [21]. Alveolar recruitment was defined as the percent of lung weight accounted for by non-aerated tissue in which aeration was restored increasing PEEP from 8 to 16 cmH_2_O, i.e.,

$${\text{Alveolar recruitment}}= \left(\frac{ {\text{Nonaerated lung tissue}}_{{\rm PEEP}\;8\;{\rm cm}\;{\rm H}_{2} {\rm O}} - {\text{Nonaerated lung tissue}}_{{\rm  PEEP}\;16\;{\rm cm}\;{\rm H}_{2}{\rm O}}}{ {\text{Total lung weight}}_{{\rm PEEP}\; 8\; {\rm cm}\;{\rm H}_{2}{\rm O}}}\right)\times 100$$

We defined patients in the fourth quartile of alveolar recruitment as “recruiters”. The lung excess lung weight was calculated as percent difference of the CT-measured lung weight at 8 cmH_2_O PEEP compared to the expected CT lung weight of a supine healthy patient, as follows:

$${\text{Excess lung weight}} \left(\%\right)= \frac{{\text{Lung weight}}_{{\rm measured},\;{\rm PEEP}\;8\;{\rm  cm}\;{\rm H}_{2}{\rm O}}-{\text{Lung  weight}}_{\rm expected}} {{\text{Lung weight}}_{\rm expected}}\times 100,$$

where ${\text{Lung weight}}_{\rm expected}\left({\text{g}}\right)=-1806.1+1633.7\times {\text{height}}\left({\text{m}}\right)$ [22]. Dynamic lung strain was calculated as the ratio of the tidal volume to the end-expiratory gas volume measured by CT scan.

### Subgroup and sensitivity analyses

To investigate the differences between phenotypes, we classified patients as *“higher compliance”* or *“lower compliance”* based on the respiratory system compliance on the day of CT scan assessed at the clinical PEEP level, with a cut-off of 40 ml/cmH_2_O, the median value reported in a recent study [11]. As a sensitivity analysis, we computed alveolar recruitment as percent change of non- and poorly-aerated compartments [21]. We also investigated the time-dependency of alveolar recruitment and respiratory system compliance exploring their correlations with the time elapsed from the onset of symptoms and initiation of invasive ventilation.

### Statistical analysis

The primary endpoint of the study was alveolar recruitment. Data are reported as median [interquartile range], if not otherwise specified. We compared data between groups with the Mann–Whitney *U* or Fisher’s exact test, as appropriate. Variables acquired at two PEEP levels were compared with the Wilcoxon signed-rank test. Correlations were sought using the Spearman’s rho. We computed median differences (MD) with their 95% confidence intervals (CI) using the Hodges–Lehman estimator. An a priori sample size calculation was not feasible due to the lack of data on quantitative CT analysis in COVID-19 patients, but our sample size was similar to previous physiologic studies in ARDS [21, 23–25]. All statistical analyses were performed in SPSS Statistics, Version 25.0 (IBM Corp., Armonk, NY, USA). Significance was assumed at two-tailed *p* < 0.05.

## Results

### Population description

Of 88 patients invasively ventilated in the study period, 42 received a two-PEEP CT scan and were included in this analysis (patient inclusion flow in the Additional file 1, eFigure 1); clinical characteristics on the day of CT are reported in Table 1. Gas-exchange and respiratory mechanics at both PEEP levels were analyzed in a subgroup of 20 patients, whose characteristics were comparable to those of the rest of the cohort (Additional file 1, eTable 1).

**Table 1:** Patients’ characteristics on the day of CT scan

Parameter	All (*N* = 42)	Non-recruiters (*N* = 32)	Recruiters (*N* = 10)	*p*
Age, median [IQR], years	63 [58–67]	64 [58–67]	65 [58–66]	0.782
Predicted body weight, median [IQR], kg	70 [61–73]	70 [61–71]	72 [70–75]	0.102
Body mass index, median [IQR], kg/m^2^	28 [25–31]	28 [25–31]	28 [26–29]	> 0.999
Male sex,* N* (%)	33 (78.6)	24 (75.0)	9 (90.0)	0.416
Time from symptoms onset, median [IQR], days	23 [13–28]	23 [13–29]	20 [17–25]	0.631
Time from first confirmed swab, median [IQR], days	15 [10–23]	17 [9–24]	13 [10–20]	0.738
Time from start of invasive ventilation, median [IQR], days	9 [4–13]	9 [4–14]	7 [3–11]	0.328
*Comorbidities*				
Hypertension,* N* (%)	24 (57.1)	18 (56.3)	6 (60.0)	> 0.999
Cardiovascular disease,* N* (%)	5 (11.9)	3 (9.4)	2 (20.0)	0.577
Smoker,* N* (%)	1 (2.4)	1 (3.1)	0 (0.0)	> 0.999
Former smoker,* N* (%)	5 (11.9)	5 (15.6)	0 (0.0)	0.315
Chronic kidney failure,* N* (%)	1 (2.4)	1 (3.1)	0 (0.0)	> 0.999
Diabetes,* N* (%)	5 (11.9)	5 (15.6)	0 (0.0)	0.315
Obesity,* N* (%)	11 (26.2)	8 (25.0)	3 (30.0)	> 0.999
*Ventilator settings*				
Tidal volume, median [IQR], ml/kg PBW	7.2 [6.3–7.9]	7.3 [6.3–8.0]	6.9 [6.2–7.6]	0.494
Respiratory rate, median [IQR], 1/min	19 [17–25]	18 [16–25]	22 [19–24]	0.273
PEEP, median [IQR], cmH_2_O	10 [8–12]	10 [8–12]	10 [8–13]	0.988
Plateau pressure, median [IQR], cmH_2_O	24 [21–28]	24 [22–28]	24 [20–27]	0.782
FiO_2_, median [IQR]	0.60 [0.50–0.70]	0.68 [0.60–0.70]	0.55 [0.50–0.65]	0.138
Respiratory system compliance, median [IQR], ml/cmH_2_O	36 [29–50]	35 [29 –50]	40 [35–45]	0.631
*Blood gas analysis*				
pH, median [IQR]	7.43 [7.36–7.48]	7.42 [7.35–7.47]	7.46 [7.43–7.49]	0.052
PaO_2_, median [IQR], mmHg	73 [64–91]	73 [65–92]	69 [64–86]	0.475
PaCO_2_, median [IQR], mmHg	48 [43–56]	51 [44–58]	41 [36–51]	0.052
PaO_2_/FiO_2_, median [IQR], mmHg	123 [100–160]	123 [98–155]	139 [103–205]	0.494
Lactate, median [IQR], mmol/L	1.1 [0.8–1.8]	1.2 [0.8–2.0]	1.1 [1.0–1.6]	0.782
Ventilatory ratio	1.8 [1.5–2.4]	2.0 [1.5–2.6]	1.8 [1.6–1.8]	0.494
*Blood analyses*				
D-dimer, median [IQR], ug/L	1647 [1048–4426]	1618 [938–4195]	1754 [1304–4426]	0.695
C reactive protein, median [IQR], mg/L	42 [17–108]	44 [19–110]	41 [17–60]	0.531
Procalcitonin, median [IQR], ug/L	0.21 [0.09–0.97]	0.25 [0.09–1.44]	0.21 [0.07–0.33]	0.423
Interleukin-6, median [IQR], ng/L	127 [55–387]	134 [55–339]	112 [16–453]	0.873
Creatinine, median [IQR], mg/dL	0.9 [0.7–1.7]	0.9 [0.6–1.7]	1.0 [0.7–1.5]	0.652
*Hemodynamics*				
Heart rate, median [IQR], 1/min	82 [70–100]	85 [72–103]	73 [68–90]	0.494
Mean arterial pressure, median [IQR], mmHg	82 [73–93]	83 [77–102]	80 [73–85]	0.551
Ventilator-associated pneumonia,* N* (%)	12 (28.6)	11 (34.3)	1 (10.0)	0.233

Gas exchange and ventilator settings measured at the clinical PEEP level

*IQR* interquartile range, *PBW* predicted body weight, *PEEP* positive end-expiratory pressure, *ICU* intensive care unit

### Alveolar recruitment and effects of PEEP on CT parameters

**Fig. 1:**
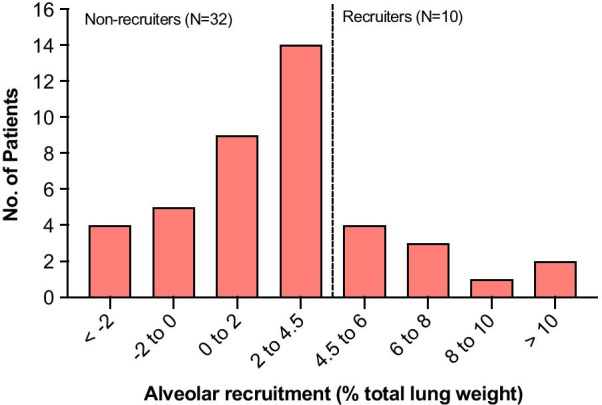
Distribution of alveolar recruitment. The dashed line represents the boundary of the third quartile (4.5%), defining the “recruiters” group

**Fig. 2:**
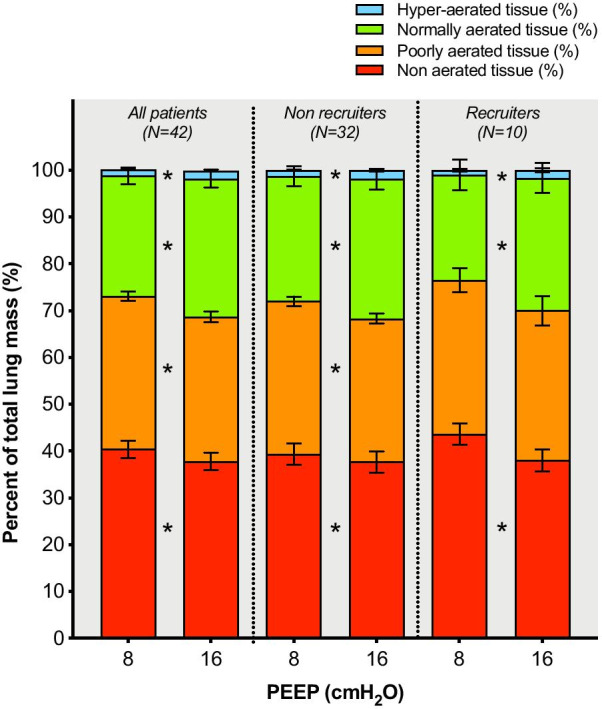
Distribution of aeration compartments, expressed as percent of the total lung mass, at PEEP of 8 and 16 cmH_2_O. Data are reported overall and stratified in the recruiter and non-recruiter groups. Bars represent means, error bars the standard error of mean. *Significant difference between the two PEEP levels (*p* < 0.05). PEEP: positive end-expiratory pressure

**Fig. 3:**
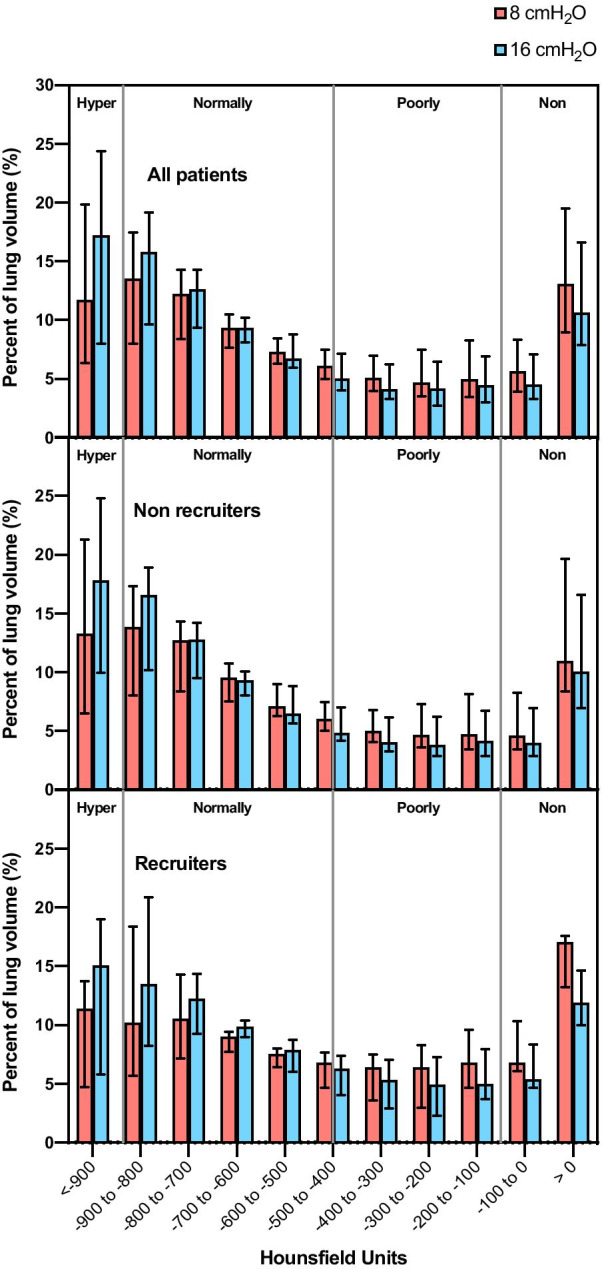
Histogram distribution of lung volume aeration along the Hounsfield units scale at the two PEEP levels. Data are reported overall (panel A) and stratified in the recruiter (panel B) and non-recruiter (panel C) groups. PEEP: positive end-expiratory pressure. Bars represent medians, error bars the interquartile ranges

Alveolar recruitment was 2.7 [0.7–4.5] % of the total lung weight or 39 [9–81] g; its distribution is reported in Fig. 1. Excess lung weight was 57 [24–75] % or 528 [240–818] g and was correlated with the amount of non-aerated tissue (*ρ* = 0.607, *p* < 0.001—Additional file 1, eFigure 2). Ten patients were classified as “recruiters”, having alveolar recruitment above the third quartile (4.5%). We did not identify differences in the clinical characteristics of recruiters vs. non-recruiters (Table 1). Increasing PEEP from 8 to 16 cmH_2_O resulted in a modest reduction in non-aerated tissue, paralleled by an increase in normally aerated and hyper-aerated tissue in both groups, while poorly aerated tissue decreased only in non-recruiters (Fig. 2 and Table 2). Figure 3 shows the distribution of lung aeration along the Hounsfield units scale at both PEEP levels. Non-aerated areas were predominantly located in dorsal and caudal regions (Additional file 1, eFigure 3). Lung dynamic strain was low and was further decreased by increasing PEEP (Table 2). The median PEEP-induced increase in lung gas content, proportional to the increase in lung static strain, was 403 ml (95% CI from 348 to 458 ml). Alveolar recruitment was not associated with disease severity as assessed by excess lung weight, PaO_2_/FiO_2_ ratio or respiratory system compliance (Additional file 1, eFigure 4). We did not observe correlations between alveolar recruitment or excess lung mass and inflammatory and thrombophilia markers (Additional file 1, eTable 2). Alveolar recruitment defined as change in the sum of poorly- and non-aerated compartments was 4.3 [2.9–6.1] % of the total lung weight and no differences were observed in lower vs. higher compliance groups (4.2 [2.6–6.0] % vs. 4.4 [3.6–6.6] %, *p* = 0.402).

**Table 2:** Quantitative CT analysis parameters

Parameter	All (*N* = 42)			Non-recruiters (*N*=32)			Recruiters (*N*=10)		
	PEEP 8 cmH_2_O	PEEP 16 cmH_2_O	*p*	PEEP 8 cmH_2_O	PEEP 16 cmH_2_O	*p*	PEEP 8 cmH_2_O	PEEP 16 cmH_2_O	*p*
Total lung volume (ml)	3076 [2610–3810]	3461 [2982–4190]	<0.001*	3120 [2687–3727]	3575 [3107–4150]	<0.001*	2619 [2331–3907]	2953 [2454–4645]	0.005*
Total lung weight (g)	1515 [1295–1811]	1539 [1336–1852]	0.866	1504 [1274–2091]	1520 [1322–2126]	0.080	1532 [1302–1773]	1455 [1244–1728]	0.116
Excess lung weight (%)	56.6 [24.0–74.8]			56.7 [23.2–90.5]			55.0 [26.3–59.59]		
Gas volume (ml)	1360 [1064–2118]	1858 [1301–2599]	<0.001*	1448 [1105–2159]	1931 [1459–2583]	<0.001*	1216 [701–2115]	1540 [1085–2957]	0.005*
Mean attenuation (HU)	-526 [-591–-329]	-565 [-637–-404]	<0.001*	-549 [-616–-333]	-581 [-644–-405]	<0.001*	-434 [-541–-301]	-539 [-634–-379]	0.005*
Hyper-aerated mass (g)	14 [7–28]	23 [12–39]	<0.001*	17 [8–29]	26 [13–39]	<0.001*	12 [5–17]	17 [8–43]	0.005*
Normally aerated mass (g)	367 [259–465]	424 [347–528]	<0.001*	375 [307–462]	440 [366–532]	<0.001*	331 [190.9–504.4]	383 [267.4–520.3]	0.005*
Poorly aerated mass (g)	504 [391–671]	487 [346–673]	<0.001*	504 [388–687]	496 [349–683]	0.001*	477 [393.9–587.5]	433 [328.1–580.8]	0.093
Non aerated mass (g)	625 [377–810]	576 [354–775]	<0.001*	564 [375–785]	581 [350–854]	0.014*	737 [515.7–819.1]	573 [435.2–710.4]	0.005*
Dynamic lung strain	0.33 [0.23–0.46]	0.26 [0.19–0.35]	<0.001*	0.33 [0.24–0.43]	0.25 [0.19–0.3]	<0.001*	0.41 [0.22–0.63]	0.33 [0.16–0.45]	<0.005*

Data are presented as median [interquartile range]

*PEEP* positive end-expiratory pressure, *HU* Hounsfield Units, *IQR* interquartile range, *CI* confidence interval

*Signigicant difference between PEEP levels, *p* < 0.05

### Data on gas exchange and respiratory mechanics at two PEEP levels

**Fig. 4:**
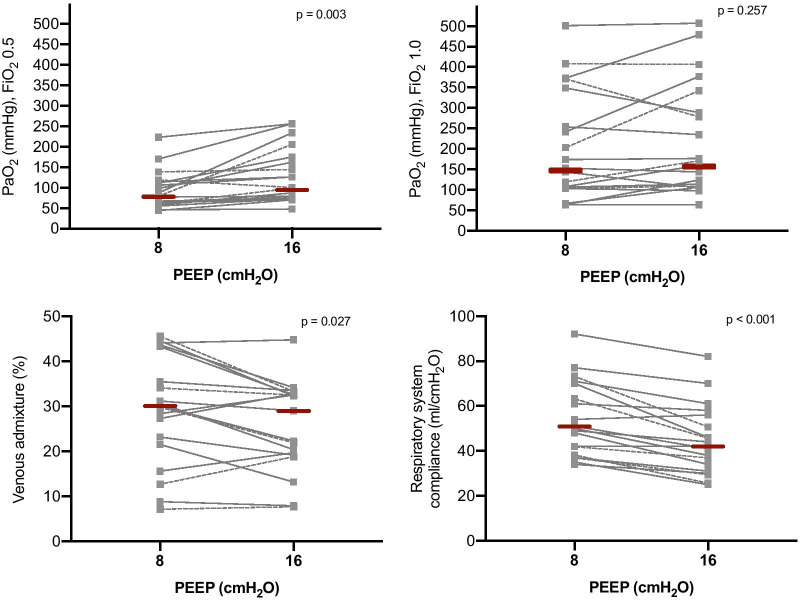
Gas-exchange and respiratory mechanics data at PEEP of 8 and 16 cmH_2_O. Gray squares and lines represent individual patient data, red bars the median value. Dashed lines represent patients in the recruiter group, solid lines the non-recruiter group. PEEP: positive end-expiratory pressure

As illustrated in Fig. 4, in patients with available data on gas-exchange and respiratory mechanics at two PEEP levels (N = 20), incrementing PEEP from 8 to 16 cmH_2_O increased the PaO_2_ when FiO_2_ = 0.5 (MD 24 mmHg, 95% CI from 12 to 51 mmHg, *p* = 0.003), but not when FiO_2_ = 1.0 (MD 7 mmHg, 95% CI from − 12 to 49 mmHg, *p* = 0.257). Maintaining PEEP at 8 cmH_2_O, increasing FiO_2_ from 0.5 to 1.0 increased the PaO_2_ (MD 103 mmHg, 95% CI from 55 to 156 mmHg, *p* < 0.001). Increasing PEEP from 8 to 16 cmH_2_O slightly reduced the venous admixture (MD − 3.5%, 95% CI from − 6.2% to − 0.4%, *p* = 0.027) and the ventilatory ratio (MD − 0.1, 95% CI from − 0.3 to − 0.1, *p* = 0.003, Additional file 1, eFigure 5), but decreased the respiratory system compliance (MD − 9 ml/cmH_2_O, 95% CI from − 12 to − 6 ml/cmH_2_O, *p* < 0.001). Improvement of oxygenation and venous admixture were not associated with alveolar recruitment (Additional file 1, eFigure 6).

### Characteristics of patients with higher versus lower compliance

Patients in the lower compared to higher compliance group had a longer time elapsed from the onset of symptoms and were ventilated with lower tidal volumes and higher plateau pressures (Additional file 1, eTable 3). Alveolar recruitment was similar in lower vs. higher compliance groups (2.5 [0.6–4.4] % vs. 3.0 [0.8–4.7] %, *p* = 0.780). Excess lung weight was similar in lower vs. higher compliance groups (52 [21–73] % vs. 57 [28–80] %, *p* = 0.799).

### Time-dependency of alveolar recruitment and respiratory system compliance

Time from the onset of symptoms and onset of invasive ventilation was similar in recruiters versus non-recruiters (Table 1), while patients in the lower compliance group had a longer time elapsed from the first confirmed swab and onset of symptoms (Additional file 1, eTable 3). The respiratory system compliance had a negative correlation with the days elapsed from the onset of symptoms (*ρ* = − 0.407, *p* = 0.007) but not with the duration of invasive ventilation (*ρ* = − 0.134, *p* = 0.397). Alveolar recruitment did not correlate with the time elapsed from the onset of symptoms (*ρ* = 0.058, *p* = 0.716) nor with the duration of invasive ventilation (*ρ* = − 0.013, *p* = 0.935).

## Discussion

The main findings of this study were that, in critically ill mechanically ventilated patients with severe COVID-19 pneumonia, alveolar recruitment induced by changes of PEEP from 8 cmH2O to 16 cmH2O was: (1) minimal and independent of the respiratory system compliance; (2) prevalent in the dependent and caudal lung regions; (3) not correlated with the excess lung weight; and (4) not associated with changes in gas-exchange, respiratory mechanics and laboratory parameters. Higher PEEP improved oxygenation at FiO_2_ 0.5 but not 1.0 and decreased respiratory system compliance.

Patients included in the present study had severe hypoxemic respiratory failure at ICU admission and at the time of CT scan. We assessed alveolar recruitment as changes in the non-aerated compartment, using classically adopted CT attenuation thresholds [21, 23–25]. The two levels of PEEP selected in the present study, i.e., 8 and 16 cmH_2_O, were the boundaries of the range of PEEP received by most COVID-19 patients [26], and similar to previous studies investigating alveolar recruitment in ARDS, where 5 and 15 cmH_2_O were used [25]. The lower level of PEEP in our study was set at 8 cmH_2_O due to safety concerns related to the reduction in PEEP to 5 cmH_2_O in severely hypoxemic COVID-19 patients.

Spontaneously breathing healthy subjects have an average lung weight of around 930 g [22]. In our cohort, lung weight was 1500 g and end-expiratory gas volume 1360 ml, values similar to those reported in studies on ARDS not related to COVID-19 [21, 25]. The gas volume was similar to that reported in a recent study, showing that when classical ARDS was compared at similar PaO_2_/FiO_2_ or compliance, the gas volume was higher in COVID-19 [5]. This is in line with a recent study comparing twenty-seven COVID-19 patients with an historical cohort of classical ARDS [11]. However, another study comparing COVID-19 ARDS with ARDS from other causes concluded that, when patients were matched based on their PaO_2_/FiO_2_ ratio or respiratory system compliance, the two pathologies had substantial differences with potential implications to the optimal ventilator management [5]. In patients with classical ARDS, the overall lung weight is increased compared to normal patients, due to increased edema distributed along a ventral-dorsal gradient [27]. This leads to increased pressures acting on the dependent lung regions and progressive atelectasis formation [27, 28]. The application of PEEP counterbalances the effects of increased superimposed pressure on most dependent alveoli [29], keeping them open and improving respiratory system compliance and gas-exchange. The median amount of alveolar recruitment from 8 to 16 cmH_2_O PEEP in our COVID-19 cohort was less than 3% of the total lung mass. This value is lower than the lung tissue recruited from 5 to 15 cmH_2_O PEEP in classical ARDS, which ranged from 8 to 15% of lung weight [21, 25] or 21% of lung volume [23] in early studies. Moreover, these studies reported high inter-subject variability in classical ARDS, while we observed a homogeneously low recruitment potential in our cohort. In line with our results, previous studies found that PEEP-induced alveolar recruitment was lower in patients with primary as compared to a secondary insult to the lung [30]. In a study in ten COVID-19 patients measuring recruitment from 5 to 15 cmH_2_O PEEP with electric impedance tomography, the recruited lung volume was around 300 ml with high inter-individual variability [31]. However, it is difficult to compare this value to our results because of the different imaging technique adopted, analyzing only one juxta-diaphragmatic slice. The application of PEEP 16 cmH_2_O also increased hyperaeration, especially in presence of less excess lung weight, as reported in classical ARDS patients [23]. As a consequence, the increase in PEEP from 8 to 16 cmH_2_O yielded  a worsening of the respiratory system compliance in our cohort of COVID-19 patients. Alveolar recruitment was not associated with higher levels of inflammatory markers, D-dimer and respiratory system compliance.

These findings support the concept that PEEP might improve oxygenation in COVID-19 by altering the $\dot{V}/\dot{Q}$ matching in areas with low ${\dot{V}}_{A}/\dot{Q}$, rather than through recruitment. This suggests caution in applying PEEP levels higher than those strictly necessary to maintain oxygenation. We observed a decrease in respiratory system compliance among patients at more advanced stages of the disease, not reflected by increased recruitment. This is compatible with a natural history of the disease based on fibrotic mechanisms, rather than worsening of edema. Our findings suggest that COVID-19 pneumonia acts as a typical primary pneumonia [32], as also confirmed by autopsy findings, which reported injury in the alveolar epithelial cells, hyaline membrane formation, and hyperplasia of type II pneumocytes, diffuse alveolar damage and consolidation due to fibroblastic proliferation with extracellular matrix and fibrin forming clusters in airspaces and capillary vessel [33]. We speculate that, differently from classical ARDS, in COVID-19 pneumonia, the non-aerated lung regions are poorly recruitable due to the fact that they do not represent atelectasis, but alveolar spaces substituted by fibrosis and mucinous filling, cellular debris and necrotic tissue reflecting pneumo-and vascular lysis [34–36].

Some limitations of our study should be addressed. In our center, CT scan and evaluation of PEEP was routinely performed in a high proportion of patients with COVID-19 pneumonia for clinical purposes, but only when CT was indicated and in sufficiently stable patients. The main reasons for exclusion were clinical instability and need for contrast-enhanced CT. The timing of CT scans was based on clinical indication, resulting in heterogeneity of included patients, and we cannot rule out that, in a proportion of patient, bacterial co-infection might have played a role in defining the radiological findings and the response to PEEP [37]. However, this is representative of the population of a COVID-19 ICU. Only two arbitrary levels of PEEP were investigated for technical reasons and patient safety concerns. While we cannot exclude that different ventilator setting or the addition of a recruitment maneuver may have led to different results, previous studies reported that the response to PEEP at two low and moderate PEEP levels correlated with the maximal lung recruitment achievable at higher PEEP [25]. Moreover, venous admixture was estimated from central venous line blood samples, not a pulmonary artery catheter.

## Conclusions

In critically ill patients with severe COVID-19 pneumonia, increasing PEEP from 8 cmH_2_O to 16 cmH_2_O did not lead to major alveolar recruitment while worsened respiratory mechanics. This suggests limiting PEEP strictly to those levels necessary to maintain oxygenation, thus avoiding the use of higher PEEP levels. Lung imaging techniques might be considered in the next future to better assess clinical alterations in critically ill patients with COVID-19 pneumonia.

## Supplementary Information

**Additional file 1**. Study details and additional analyses.

## Data Availability

Dataset available from the corresponding author upon reasonable request.
