# Supplementary material for: Computed tomography assessment of PEEP-induced alveolar recruitment in patients with severe COVID-19 pneumonia
Source: Crit Care. 2021 Feb 24;25:81. doi: 10.1186/s13054-021-03477-w (PMC7903929; doi:10.1186/s13054-021-03477-w)
Supplement: Supplementary file 1 — Additional file 1. Study details and additional analyses. [file 13054_2021_3477_MOESM1_ESM.docx]

Computed Tomography Assessment of PEEP-induced Alveolar Recruitment in Patients with Severe COVID-19 Pneumonia

*Lorenzo Ball, Chiara Robba, Lorenzo Maiello, Jacob Herrmann, Sarah E Gerard, Yi Xin, Denise Battaglini, Iole Brunetti, Giuseppe Minetti, Sara Seitun, Antonio Vena, Daniele Roberto Giacobbe, Matteo Bassetti, Patricia RM Rocco, Maurizio Cereda, Lucio Castellan, Nicolò Patroniti, Paolo Pelosi*

Table of Contents

[Clinical context, rationale and indications for CT scan 2](#_Toc60220232)

[Protocol for the two-PEEP CT scan acquisition and analysis 2](#_Toc60220233)

[Assessment of gas exchange and respiratory mechanics 3](#_Toc60220234)

[Definition of Ventilator-Associated Pneumonia 4](#_Toc60220235)

[eFigure 1 – Patient inclusion flow 5](#_Toc60220236)

[eTable 1. Characteristics of patients with and without gas exchange data on the day of CT scan. 6](#_Toc60220237)

[eTable 2 – Associations with inflammatory and thrombophilia markers 7](#_Toc60220238)

[eTable 3. Characteristics of patients in two respiratory system compliance groups. 8](#_Toc60220239)

[eFigure 2 – Associations between lung excess mass and aeration at PEEP 8 cmH_2_O. 10](#_Toc60220240)

[eFigure 3 – Ventral-dorsal and apical-caudal ROIs 11](#_Toc60220241)

[eFigure 4 – Associations between lung recruitment and disease severity 12](#_Toc60220242)

[eFigure 5 – Ventilatory ratio at two PEEP levels 13](#_Toc60220243)

[eFigure 6 – Associations between recruitment and gas exchange 13](#_Toc60220244)

[References 14](#_Toc60220245)

# Clinical context, rationale and indications for CT scan

The first intubated COVID-19 patient was admitted to our intensive care unit (ICU) on February 29^th^, 2020, during a national emergency and in lack of clinical data concerning the disease. Most patients at admission met the criteria for moderate to severe acute respiratory distress syndrome (ARDS) and were treated according to the current clinical practice and recommendations, including the use of moderate to high positive end-expiratory pressure (PEEP) levels(1). In the first weeks, we observed a wide range of respiratory system compliance at admission and dissociation between compliance and the PaO_2_/FiO_2_ ratio, suggesting differences with classical ARDS. Moreover, the response to PEEP increase, while improving gas exchange, often resulted in worsening of the respiratory mechanics, with increased driving pressure and decreased compliance. Therefore, starting from March 20^th^ 2020, we started performing two-PEEP computed tomography (CT) as part of the clinical evaluation of patients with COVID-19 pneumonia. Images were initially evaluated visually to estimate the individual potential for lung recruitment(2), then after approval of the Ethical Review Board we started retrieving images for quantitative analysis. We performed the two-PEEP CT scan in all patients with a clinical indication for chest CT, excluding those: 1) requiring contrast medium for clinical reasons, 2) having contraindications to higher PEEP (e.g. undrained pneumothorax), 3) judged too instable to be safely transported to the CT facility.

# Protocol for the two-PEEP CT scan acquisition and analysis

Chest CT scans were performed during end-expiratory breath hold using one of the two available scanners: a Somatom Definition Flash (38 patients, Siemens, Erlangen, Germany) and an Optima CT660 (4 patients, GE Medical Systems, Little Chalfont, Buckinghamshire, United Kingdom). The former was operated at 140 kVp, the latter at 120 kVp, with dose modulation activated on both scanners. Images were reconstructed at a slice thickness of 0.75 mm (N = 36), 1.25 mm (N = 2), 2.5 mm (N = 2) or 5 mm (N = 2), using sharp lung kernels (B80f on the Siemens scanner, *Lung* kernel on the GE scanner). The lung weight was assumed proportional to the gas vs. tissue fraction contained in each voxel, approximating the tissue density as equal to the water density. (3) Segmentation of lung parenchyma was performed automatically using a multi-resolution convolutional neural network(4) with automated exclusion of airways, followed by manual refinement as necessary. Three regions of interests (ROIs) of equal lung tissue weight(3, 5) were obtained along the ventral-dorsal and craniocaudal axes. In all analyses, ROIs were further subdivided according to their degree of aeration, using standard thresholds (-900 HU, -500 HU, and -100 HU to define hyper-aerated, normal, poorly aerated, and non-aerated lung regions)(6).

# Assessment of gas exchange and respiratory mechanics

We assessed gas exchange and respiratory mechanics at PEEP 8 cmH_2_O and 16 cmH_2_O to help the clinician in deciding the individual level of PEEP. In this analysis, we included gas exchange and respiratory mechanics data of patients in which the assessment was performed within two hours from the execution of the CT scan. The assessment was performed during volume-controlled mechanical ventilation, with tidal volume, respiratory rate and FiO_2_ set as per clinical indication. Gas exchange was assessed at a FiO_2_ of 0.5 and 1.0. Blood gas analyses were performed after allowing 5 minutes for stabilization. Venous admixture was assessed at FiO_2_ 1.0 at the two PEEP levels with the following formula:

$$\frac{\dot{Q}_{VA}}{\dot{Q}_{T}}=\frac{CcO_{2}-CaO_{2}}{CcO_{2}-CvO_{2}}$$

Where the capillary oxygen content (CcO_2_) was computed as:

$${CcO}_{2}=\left( {P_{A}O}_{2}\cdot0.0031 \right)+\left[ Hb \right]\cdot100\%\cdot1.36$$

assuming

$${P_{A}O}_{2}=\left( 760 mmHg-47 mmHg \right)\cdot1-\frac{{PaCO}_{2}}{0.8}$$

The arterial oxygen content (CaO_2_) was computed as:

$${CaO}_{2}=\left( {PaO}_{2}\cdot0.0031 \right)+\left[ Hb \right]\cdot{SaO}_{2}\cdot1.36$$

The venous oxygen content (CvO_2_) was estimated from a central venous blood sample as:

$${CvO}_{2}=\left( {PvO}_{2}\cdot0.0031 \right)+\left[ Hb \right]\cdot{SvO}_{2}\cdot1.36$$

The compliance of the respiratory system was computed as:

$$C_{rs}=\frac{V_{T}}{P_{plat}-PEEP}$$

Where V_T_ is the tidal volume and P_plat_ the plateau pressure measured after a manual inspiratory hold.

# Definition of Ventilator-Associated Pneumonia

Patients were classified as having or not having ventilator-associated pneumonia (VAP) on the day of computed tomography (CT) scan based on clinical adjudication, performed by two independent infectious disease specialists, based on the following criteria: new chest X-ray infiltrates occurring more than 48 hours after initiation of invasive ventilation, plus:

1. New onset of body temperature ≥38 °C or ≤35 °C AND/OR White blood cell count ≥10,000 cells/μL or ≤4500 cells/μL

AND

1. New onset of respiratory secretions requiring suctioning AND/OR Need for increased FiO2 or PEEP to maintain oxygenation

# eFigure 1 – Patient inclusion flow


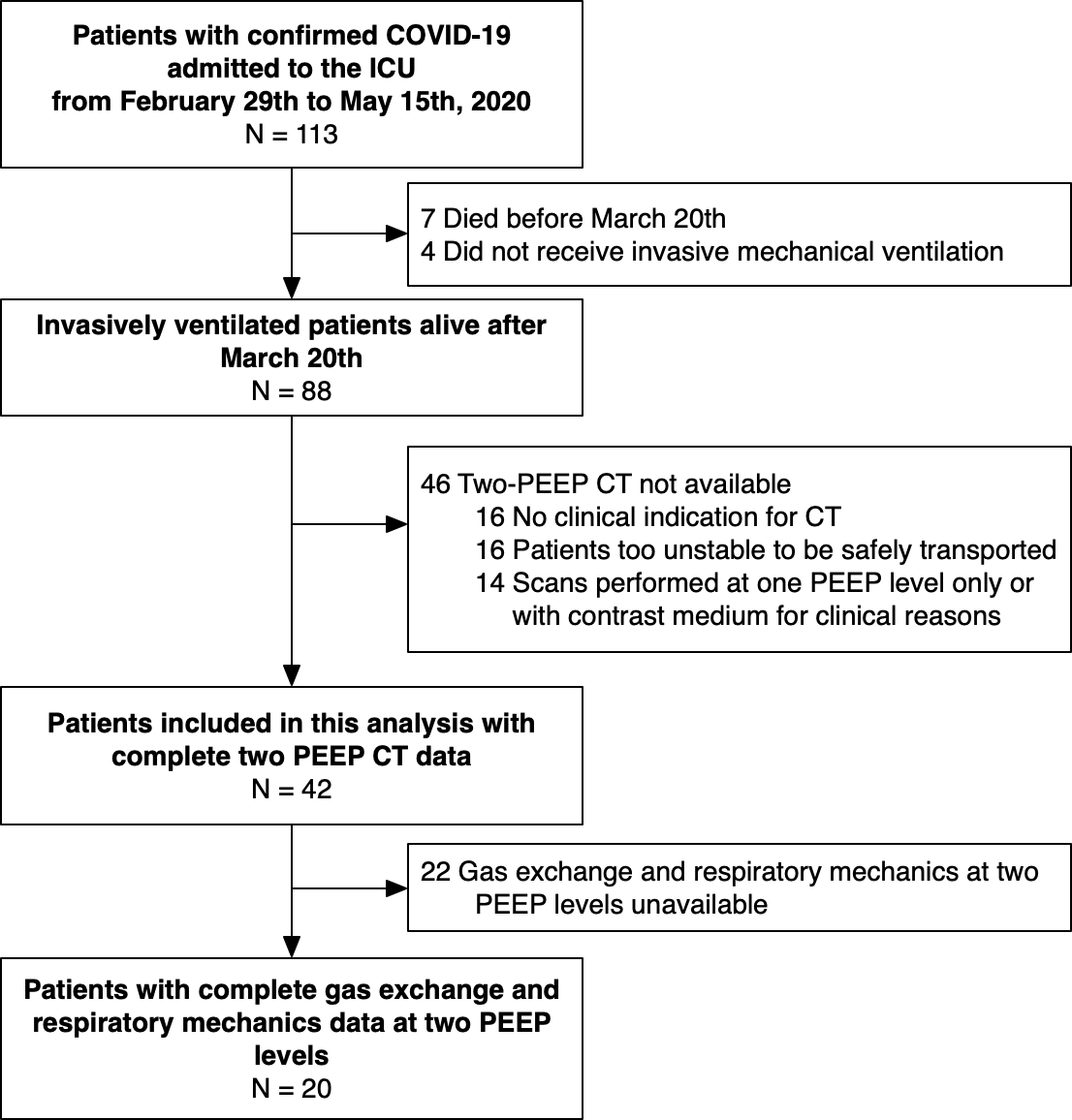


**eFigure 1**: Patient inclusion flow. PEEP: positive end-expiratory pressure; CT: computed tomography.

# eTable 1. Characteristics of patients with and without gas exchange data on the day of CT scan.

| **Parameter** | **All (N=42)** | **With gas exchange data (N=20)** | **Without gas exchange data (N=22)** | **p** |
| --- | --- | --- | --- | --- |
| Age, median [IQR], years | 63 [58 - 67] | 65 [57 - 68] | 62 [58 - 66] | 0.495 |
| Predicted body weight, median [IQR], kg | 70 [61 - 73] | 71 [66 - 75] | 68 [61 - 72] | 0.656 |
| Body mass index, median [IQR], kg/m^2^ | 28 [25 - 31] | 26 [24 - 29] | 28 [26 - 31] | 0.217 |
| Male sex, N (%) | 33 (78.6) | 17 (77.3) | 16 (80.0) | >0.999 |
| Time from symptoms onset, median [IQR], days | 23 [13 - 28] | 18 [13 - 26] | 25 [17 - 29] | 0.194 |
| Time from first confirmed swab, median [IQR], days | 15 [10 - 23] | 11 [10 - 20] | 19 [10 - 25] | 0.246 |
| Time from start of invasive ventilation, median [IQR], days | 9 [4 - 13] | 9 [4 - 12] | 9 [5 - 18] | 0.457 |
| **Comorbidities** |  |  |  |  |
| Hypertension, N (%) | 24 (57.1) | 15 (68.2) | 9 (45.0) | 0.212 |
| Cardiovascular disease, N (%) | 5 (11.9) | 5 (22.7) | 0 (0.0) | 0.049* |
| Smoker, N (%) | 1 (2.4) | 1 (4.5) | 0 (0.0) | >0.999 |
| Former smoker, N (%) | 5 (11.9) | 3 (13.6) | 2 (10.0) | >0.999 |
| Chronic kidney failure, N (%) | 1 (2.4) | 1 (4.5) | 0 (0.0) | >0.999 |
| Diabetes, N (%) | 5 (11.9) | 3 (13.6) | 2 (10.0) | >0.999 |
| Obesity, N (%) | 11 (26.2) | 7 (31.8) | 4 (20.0) | 0.491 |
| **Ventilator settings** |  |  |  |  |
| Tidal volume, median [IQR], ml/kg PBW | 7.2 [6.3 - 7.9] | 7.4 [6.5 - 7.9] | 7.1 [6.2 - 7.9] | 0.457 |
| Respiratory rate, median [IQR], 1/min | 19 [17 - 25] | 17 [16 - 23] | 21 [18 - 28] | 0.019* |
| PEEP, median [IQR], cmH_2_O | 10 [8 - 12] | 10 [8 - 12] | 10 [9 - 12] | 0.768 |
| Plateau pressure, median [IQR], cmH_2_O | 24 [21 - 28] | 24 [21 - 28] | 24 [22 - 28] | 0.705 |
| FiO_2_, median [IQR] | 0.60 [0.50 - 0.70] | 0.55 [0.40 - 0.65] | 0.70 [0.60 - 0.70] | 0.003* |
| Respiratory system compliance, median [IQR], ml/cmH_2_O | 36 [29 - 50] | 37 [30 - 48] | 35 [29 - 50] | 0.605 |
| **Blood gas analysis** |  |  |  |  |
| pH, median [IQR] | 7.43 [7.36 - 7.48] | 7.44 [7.41 - 7.48] | 7.41 [7.35 - 7.46] | 0.173 |
| PaO_2_, median [IQR], mmHg | 73 [64 - 91] | 71 [62 - 81] | 75 [67 - 94] | 0.155 |
| PaCO_2_, median [IQR], mmHg | 48 [43 - 56] | 45 [42 - 56] | 51 [43 - 56] | 0.450 |
| PaO_2_/FiO_2_, median [IQR], mmHg | 123 [100 - 160] | 135 [113 - 175] | 110 [96 - 159] | 0.174 |
| Lactate, median [IQR], mmol/L | 1.1 [0.8 - 1.8] | 1.3 [0.8 - 2.1] | 1.1 [0.8 - 1.4] | 0.425 |
| Ventilatory ratio | 1.8 [1.5 – 2.4] | 1.7 [1.3 – 2.2] | 1.9 [1.6 – 2.6] | 0.121 |
| **Blood analyses** |  |  |  |  |
| D-dimer, median [IQR], ug/L | 1647 [1048 - 4426] | 2584 [1101 - 12074] | 1480 [835 - 1893] | 0.134 |
| C reactive protein, median [IQR], mg/L | 42 [17 - 108] | 25 [10 - 66] | 52 [21 - 111] | 0.162 |
| Procalcitonin, median [IQR], ug/L | 0.21 [0.09 - 0.97] | 0.17 [0.07 - 0.58] | 0.26 [0.10 - 1.22] | 0.420 |
| Interleukin-6, median [IQR], ng/L | 127 [55 - 387] | 161 [93 - 452] | 84 [16 - 199] | 0.064 |
| Creatinine, median [IQR], mg/dL | 0.9 [0.7 - 1.7] | 1.0 [0.7 - 1.4] | 0.8 [0.7 - 1.7] | 0.781 |
| **Hemodynamics** |  |  |  |  |
| Heart rate, median [IQR], 1/min | 82 [70 - 100] | 79 [68 - 94] | 85 [71 - 111] | 0.212 |
| Mean arterial pressure, median [IQR], mmHg | 82 [73 - 93] | 79 [72 - 98] | 83 [78 - 93] | 0.465 |
| Ventilator-associated pneumonia, N (%) | 12 (28.6) | 8 (40.0) | 4 (18.2) | 0.315 |

PEEP: positive end-expiratory pressure; ICU: intensive care unit; PBW: predicted body weight.

# eTable 2 – Associations with inflammatory and thrombophilia markers

|  | **Lung recruitment (%)** | **Excess lung weight (%)** |
| --- | --- | --- |
| D-dimer (ug/L) | ρ = -0.051, p = 0.750 | ρ = 0.049, p = 0.759 |
| C reactive protein (mg/L) | ρ = 0.152, p = 0.335 | ρ = 0.072, p = 0.651 |
| Procalcitonin (ug/L) | ρ = 0.190, p = 0.229 | ρ = -0.064, p = 0.689 |
| Interleukin-6 (ng/L) | ρ = -0.142, p = 0.371 | ρ = 0.013, p = 0.933 |
| Respiratory system compliance (ml/cmH_2_O) | ρ = -0.068, p = 0.667 | ρ = -0.068, p = 0.668 |
| PaO_2_/FiO_2_ | ρ = 0.035, p = 0.828 | ρ = -0.296, p = 0.057 |

# eTable 3. Characteristics of patients in two respiratory system compliance groups.

| **Parameter** | **All (N=42)** | **Lower compliance (N=24)** | **Higher compliance (N=18)** | **p** |
| --- | --- | --- | --- | --- |
| Age, median [IQR], years | 63 [58 - 67] | 64 [58 - 67] | 62 [60 - 66] | 0.779 |
| Predicted body weight, median [IQR], kg | 70 [61 - 73] | 70 [61 - 75] | 70 [61 - 71] | 0.990 |
| Body mass index, median [IQR], kg/m^2^ | 28 [25 - 31] | 28 [26 - 31] | 28 [25 - 31] | >0.99 |
| Male sex, N (%) | 33 (78.6) | 18 (75.0) | 15 (83.3) | 0.708 |
| Time from symptoms onset, median [IQR], days | 23 [13 - 28] | 26 [17 - 37] | 17 [13 - 25] | 0.065 |
| Time from first confirmed swab, median [IQR], days | 15 [10 - 23] | 20 [11 - 30] | 10 [9 - 18] | 0.025* |
| Time from start of invasive ventilation, median [IQR], days | 9 [4 - 13] | 11 [4 - 13] | 7 [4 - 13] | 0.315 |
| **Comorbidities** |  |  |  |  |
| Hypertension, N (%) | 24 (57.1) | 13 (54.2) | 11 (61.1) | 0.757 |
| Cardiovascular disease, N (%) | 5 (11.9) | 3 (12.5) | 2 (11.1) | >0.99 |
| Smoker, N (%) | 1 (2.4) | 1 (4.2) | 0 (0.0) | >0.99 |
| Former smoker, N (%) | 5 (11.9) | 3 (12.5) | 2 (11.1) | >0.99 |
| Chronic kidney failure, N (%) | 1 (2.4) | 1 (4.2) | 0 (0.0) | >0.99 |
| Diabetes, N (%) | 5 (11.9) | 5 (20.8) | 0 (0.0) | 0.060 |
| Obesity, N (%) | 11 (26.2) | 5 (20.8) | 6 (33.3) | 0.483 |
| **Ventilator settings** |  |  |  |  |
| Tidal volume, median [IQR], ml/kg PBW | 7.2 [6.3 - 7.9] | 6.9 [6.1 - 7.6] | 7.7 [7.2 - 8.9] | 0.008* |
| Respiratory rate, median [IQR], 1/min | 19 [17 - 25] | 21 [16 - 26] | 18 [17 - 22] | 0.507 |
| PEEP, median [IQR], cmH_2_O | 10 [8 - 12] | 10 [8 - 12] | 10 [10 - 13] | 0.127 |
| Plateau pressure, median [IQR], cmH_2_O | 24 [21 - 28] | 27 [24 - 29] | 21 [19 - 25] | <0.001* |
| FiO_2_, median [IQR] | 0.60 [0.50 - 0.70] | 0.60 [0.50 - 0.70] | 0.63 [0.50 - 0.70] | 0.907 |
| Respiratory system compliance, median [IQR], ml/cmH_2_O | 36 [29 - 50] | 30 [22 - 35] | 50 [44 - 63] | <0.001* |
| **Blood gas analysis** |  |  |  |  |
| pH, median [IQR] | 7.43 [7.36 - 7.48] | 7.43 [7.35 - 7.47] | 7.43 [7.37 - 7.48] | 0.532 |
| PaO_2_, median [IQR], mmHg | 73 [64 - 91] | 72 [66 - 88] | 75 [64 - 94] | 0.949 |
| PaCO_2_, median [IQR], mmHg | 48 [43 - 56] | 51 [41 - 62] | 47 [43 - 52] | 0.374 |
| PaO_2_/FiO_2_, median [IQR], mmHg | 123 [100 - 160] | 122 [94 - 165] | 128 [107 - 160] | 0.684 |
| Lactate, median [IQR], mmol/L | 1.1 [0.8 - 1.8] | 1.2 [1.0 - 1.8] | 1.0 [0.8 - 1.8] | 0.220 |
| Ventilatory ratio | 1.8 [1.5 – 2.4] | 1.9 [1.2 – 2.6] | 1.8 [1.6 – 2.2] | 0.799 |
| **Blood analyses** |  |  |  |  |
| D-dimer, median [IQR], ug/L | 1647 [1048 - 4426] | 1471 [831 - 3962] | 1684 [1050 - 6866] | 0.576 |
| C reactive protein, median [IQR], mg/L | 42 [17 - 108] | 29 [17 - 66] | 62 [25 - 136] | 0.118 |
| Procalcitonin, median [IQR], ug/L | 0.21 [0.09 - 0.97] | 0.37 [0.10 - 1.44] | 0.13 [0.09 - 0.46] | 0.186 |
| Interleukin-6, median [IQR], ng/L | 127 [55 - 387] | 130 [49 - 373] | 127 [55 - 387] | 0.839 |
| Creatinin, median [IQR], mg/dL | 0.9 [0.7 - 1.7] | 1.1 [0.6 - 1.7] | 0.9 [0.7 - 1.1] | 0.730 |
| **Hemodynamics** |  |  |  |  |
| Heart rate, median [IQR], 1/min | 82 [70 - 100] | 94 [80 - 111] | 72 [66 - 81] | 0.002* |
| Mean arterial pressure, median [IQR], mmHg | 82 [73 - 93] | 80 [70 - 90] | 84 [78 - 103] | 0.088 |
| Ventilator-associated pneumonia, N (%) | 12 (28.6) | 9 (37.5) | 3 (16.7) | 0.180 |

Patients’ characteristics on the day of CT scan according to their respiratory system compliance (cut-off value 40 ml/cmH_2_O). IQR: interquartile range; PBW predicted body weight; PEEP: Positive End-Expiratory Pressure; ICU: intensive care unit.

# eFigure 2 – Associations between lung excess mass and aeration at PEEP 8 cmH_2_O.

**eFigure 2:** Associations between lung excess mass and aeration at PEEP 8cmH_2_O.

# eFigure 3 – Ventral-dorsal and apical-caudal ROIs

**eFigure 3:** Lung aeration compartments in three ventral-to-dorsal and three apical-to-caudal regions of equal mass at PEEP level of 8 cmH_2_O and 16 cmH_2_O.

# eFigure 4 – Associations between lung recruitment and disease severity

**eFigure 4:** Associations between lung recruitment and disease severity.

# eFigure 5 – Ventilatory ratio at two PEEP levels

**eFigure 5:** Ventilatory ratio at PEEP of 8 and 16 cmH_2_O. Gray squares and lines represent individual patient data, red bars the median value. Dashed lines represent patients in the recruiters group, solid lines the non-recruiters group. PEEP: positive end-expiratory pressure.

# eFigure 6 – Associations between recruitment and gas exchange

**eFigure 6:** Associations between recruitment and gas exchange. The venous admixture is estimated with FiO_2_ of 1.0.

# References

1. Fan E, Del Sorbo L, Goligher EC, Hodgson CL, Munshi L, Walkey AJ, Adhikari NKJ, Amato MBP, Branson R, Brower RG, Ferguson ND, Gajic O, Gattinoni L, Hess D, Mancebo J, Meade MO, McAuley DF, Pesenti A, Ranieri VM, Rubenfeld GD, Rubin E, Seckel M, Slutsky AS, Talmor D, Thompson BT, Wunsch H, Uleryk E, Brozek J, Brochard LJ, *et al.* An Official American Thoracic Society/European Society of Intensive Care Medicine/Society of Critical Care Medicine Clinical Practice Guideline: Mechanical Ventilation in Adult Patients with Acute Respiratory Distress Syndrome. *Am J Respir Crit Care Med* 2017;195:1253–1263.

2. Chiumello D, Marino A, Brioni M, Menga F, Cigada I, Lazzerini M, Andrisani MC, Biondetti P, Cesana B, Gattinoni L. Visual anatomical lung CT scan assessment of lung recruitability. *Intensive Care Med* 2013;39:66–73.

3. Protti A, Iapichino GE, Milesi M, Melis V, Pugni P, Comini B, Cressoni M, Gattinoni L. Validation of computed tomography for measuring lung weight. *Intensive Care Med Exp* 2014;2:31.

4. Gerard SE, Herrmann J, Kaczka DW, Musch G, Fernandez-Bustamante A, Reinhardt JM. Multi-resolution convolutional neural networks for fully automated segmentation of acutely injured lungs in multiple species. *Med Image Anal* 2020;60:101592.

5. Güldner A, Braune A, Ball L, Silva PL, Samary C, Insorsi A, Huhle R, Rentzsch I, Becker C, Oehme L, Andreeff M, Vidal Melo MF, Winkler T, Pelosi P, Rocco PRM, Kotzerke J, Gama de Abreu M. Comparative Effects of Volutrauma and Atelectrauma on Lung Inflammation in Experimental Acute Respiratory Distress Syndrome. *Crit Care Med* 2016;44:e854-865.

6. Ball L, Brusasco C, Corradi F, Paparo F, Garlaschi A, Herrmann P, Quintel M, Pelosi P. Lung hyperaeration assessment by computed tomography: correction of reconstruction-induced bias. *BMC Anesthesiol* 2016;16:67.
